# Integrin-Alpha IIb Identifies Murine Lymph Node Lymphatic Endothelial Cells Responsive to RANKL

**DOI:** 10.1371/journal.pone.0151848

**Published:** 2016-03-24

**Authors:** Olga G. Cordeiro, Mélanie Chypre, Nathalie Brouard, Simon Rauber, Farouk Alloush, Monica Romera-Hernandez, Cécile Bénézech, Zhi Li, Anita Eckly, Mark C. Coles, Antal Rot, Hideo Yagita, Catherine Léon, Burkhard Ludewig, Tom Cupedo, François Lanza, Christopher G. Mueller

**Affiliations:** 1 CNRS UPR 3572, University of Strasbourg, Laboratory of Immunopathology and Therapeutic Chemistry/ MEDALIS, Institut de Biologie Moléculaire et Cellulaire, Strasbourg, France; 2 Prestwick Chemical, Blvd Gonthier d'Andernach, Parc d’innovation, 67400, Illkirch, France; 3 INSERM, UMR_S949, Etablissement Français du Sang-Alsace, Faculté de Médecine, Fédération de Médecine Translationnelle, Université de Strasbourg, Strasbourg, France; 4 Department of Hematology, Erasmus University Medical Center, Rotterdam, The Netherlands; 5 BHF Centre for Cardiovascular Science, Queens Medical Research Institute, University of Edinburgh, Edinburgh, United Kingdom; 6 Center for Immunology and Infection, Department of Biology, University of York, York, United Kingdom; 7 Department of Immunology, Juntendo University School of Medicine, Tokyo, 113–8421, Japan; 8 Institute of Immunobiology, Kantonspital St. Gallen, 9007, St. Gallen, Switzerland; McGill University, CANADA

## Abstract

Microenvironment and activation signals likely imprint heterogeneity in the lymphatic endothelial cell (LEC) population. Particularly LECs of secondary lymphoid organs are exposed to different cell types and immune stimuli. However, our understanding of the nature of LEC activation signals and their cell source within the secondary lymphoid organ in the steady state remains incomplete. Here we show that integrin alpha 2b (ITGA2b), known to be carried by platelets, megakaryocytes and hematopoietic progenitors, is expressed by a lymph node subset of LECs, residing in medullary, cortical and subcapsular sinuses. In the subcapsular sinus, the floor but not the ceiling layer expresses the integrin, being excluded from ACKR4^+^ LECs but overlapping with MAdCAM-1 expression. ITGA2b expression increases in response to immunization, raising the possibility that heterogeneous ITGA2b levels reflect variation in exposure to activation signals. We show that alterations of the level of receptor activator of NF-κB ligand (RANKL), by overexpression, neutralization or deletion from stromal marginal reticular cells, affected the proportion of ITGA2b^+^ LECs. Lymph node LECs but not peripheral LECs express RANK. In addition, we found that lymphotoxin-β receptor signaling likewise regulated the proportion of ITGA2b^+^ LECs. These findings demonstrate that stromal reticular cells activate LECs via RANKL and support the action of hematopoietic cell-derived lymphotoxin.

## Introduction

Molecules, cells and pathogens carried by the lymph flow are filtered by lymph nodes (LNs). In these specialized organs, resident immune cells recognize, eliminate and mount an immune response against pathogens. The LECs provide an important structural and functional support to this process by mediating lymph drainage, organizing cellular compartments, regulating the immune response and controlling lymph exit [1]. Lymph first drains into the subcapsular sinus, which comprises an outermost (ceiling-lining) and an inner (floor-lining) lymphatic endothelial layer. Differential expression of the chemokine ACKR4 (also called CCRL1) has recently highlighted structural and functional specialization of these layers [2]. LECs also form the cortical and medullary sinuses that allow distribution of cells and large molecules within different LN compartments and exit into the efferent lymph [3]. Platelet adhesion to lymphatic endothelium mediates blood and lymphatic vessel separation during embryonic development [4].

Integrins play an important role in a variety of biological processes ranging from development, cancer, and inflammation [5]. The large family of transmembrane receptors, composed of α and β subunits, provides structural and functional integrity to connective tissues and organs, mediates cell extravasation from blood and contributes to cell activation. The integrin α2b (ITGA2b, CD41 or glycoprotein IIb) pairs exclusively with integrin β3 (ITGB3, CD61 or glycoprotein IIIa), while the latter can also form a heterodimer with integrin αV (ITGAV, CD51). ITGA2B/ITGB3 is well known for its role in blood clotting through its expression by megakaryocytes and platelets [6]. Upon platelet stimulation, the surface integrin heterodimer becomes activated, binds fibrinogen and von Willebrand factor resulting in platelet aggregation. ITGA2b and ITGB3 are also expressed by embryonic erythroid and hematopoietic progenitor cells arising from the hemogenic endothelium of the conceptus and embryo [7–9]. Although hemogenic endothelium generates ITGA2b^+^ hematopoietic progenitor cells, these special endothelial cells themselves lack the integrin [7]. Otherwise, blood endothelial cells express a number of integrins, both in the abluminal space to adhere to the basement membrane and in the lumen to recruit leucocytes [5].

The TNF family member RANKL (TNFSF11), alike other member of the protein family such as lymphotoxin-α and β, plays an important role in LN development [10]. It is expressed in the embryo by the hematopoietic lymphoid tissue inducing cells and triggers lymphotoxin production [11]. In a second phase RANKL is expressed by the lymphoid organizer cells of mesenchymal origin [12], which are thought to persist as marginal reticular cells (MRCs) in the adult [13]. The role of RANKL produced by MRCs remains unknown. In a model of skin overexpression RANKL was shown to activate LN lymphatic and blood endothelial cells as well as fibroblastic reticular cells raising the possibility that RANKL of MRCs functions as internal activator of these cells [14].

In this study, we show that a subset of LECs of mouse and human LNs express ITGA2b. In the murine LN the ITGA2b^+^ LECs are heterogeneously distributed in the medullary and cortical areas as well as in the subcapsular sinus, where only the floor-lining cells carry the integrin. ITGA2b could potentially heterodimerize with ITGB3 to bind ligands, such as fibronectin, but the alternative α-chain, ITGAV, is also present to pair with ITGB3 to anchor the cells to matrix components. In mice overexpressing RANKL the level of ITGA2b increases, while its neutralization or its genetic deletion from MRCs reduce the integrin expression. Similarly, inhibition of lymphotoxin-β receptor signaling negatively affects the proportion of ITGA2b^+^ LECs. Therefore, ITGA2b is a novel marker for LN LECs constitutively activated by TNF-family members RANKL and lymphotoxin-αβ.

## Material and Methods

### Mice

C57BL/6, *Itga2b*^-/-^ [15], ACKR4-eGFP transgenic mice (otherwise known as CCRL-1-eGFP) [2], RANK-transgenic [14], and RANKL^ΔCcl19^ mice were bred and kept in specific pathogen-free conditions, and all experiments were carried out in conformity to the animal bioethics legislation approved by and according to national guidelines of the CREMEAS (Comité Régional d’Ethique en Matière d’Expérimentation Animale de Strasbourg), permit number AL/02/22/11/11 and AL/03/12/05/12. All efforts were made to minimize suffering. To generate mice with conditional RANKL deficiency in marginal reticular cells (*RANKL*^*ΔCcl19*^), mice containing a single copy of the *Ccl19*-*cre* BAC transgene [16] were crossed with RANKL^*f/f*^ (B6.129-Tnfsf11tm1.1Caob/J) mice [17]. For adoptive bone marrow transfer, 6-wk-old mice were lethally irradiated with 9 Gy (Caesium source), and 3 h later they received 5x10^6^ bone marrow cells i.v. harvested from *Itga2b*^-/-^ mice. Chimerism was complete by the absence of ITGA2b on platelets.

### Preparation of LN Stromal Cells

Stromal cells were prepared from murine peripheral (inguinal, axial and brachial) or mesenteric LNs as previously described [18]. CD45^+^ and TER119^+^ cells were depleted using anti-TER119 and anti-CD45 coupled magnetic beads (Miltenyi Biotec). Use of all human tissues was approved by the Medical Ethical Commission of the Erasmus University Medical Center Rotterdam and was contingent on written informed consent from the donor. Stromal cells from human LNs were obtained as described [19].

### Flow Cytometry and Cell Sorting

All reactions were performed at 4°C for 20 min in PBS supplemented with 2% FCS and 2.5 mM EDTA. The following antibodies were used for flow cytometry: CD45-APC/CY7 (30-F11, Biolegend), Ter119-APC/CY7 (Ter119, Biolegend), gp38/podoplanin-A488 (8.1.1, Biolegend), CD31-PcPeF710 (390, eBioscience), ITGA2b (APC-conjugated MWReg30, Biolegend, A647-conjugated RAM-2), ITGB3-PE (2C9.G2, Biolegend), glycoprotein subunit IBβ-A647 (RAM-1 [20]), ITGAV-PE (RMV-7, eBioscience), CD3-FITC (145-2C11, BD), CD19-APC (1D3, BD), CD103-PerCP-Cy5.5 (M290, BD), CD11c-PerCP-Cy5.5 (N418, BD), RANK-02 [21] or their isotype controls. Integrin αIIbβ3-PE (JON/A, EMFRET analytics GmbH, Eibelstadt, Germany) was used to stain for the active integrin conformation in tyrode-albumin buffer pH 7.3 (137 mM NaCl, 2,7mM KCl, 12mM NaHCO3, 0.36 mM NaH2PO4, 1mM MgCl2, 2mM CaCl2, 5mM Hepes, 0,35% albumin, 5.55 mM Glucose). Flow cytometry was performed on a Gallios (Beckman-Coulter, Fullerton, CA, USA) or a Fortessa X-20 SORP (BD) and analyzed with FlowJo software (Treestar, Ashland, OR, USA). For flow cytometric analysis of human fetal LNs the following antibodies were used: gp38/podoplanin A488 (NC-08, Biolegend), CD31 Pacific-blue (WM59, Biolegend), CD45 PE-Cy7 (HI30, Biolegend), and mouse anti-Donkey A647 (Life technologies). Primary antibodies were added to the cells for 30 min at 4ᵒC. Then, cells were stained with secondary antibodies for 20 min at 4°C.

### Lymphatic Endothelial Cell Culture

LECs were cell-sorted based on gp38 and CD31 expression and cultured in a single drop of endothelial cell growth medium (Lonza) in culture slides (Corning) pre-coated with 5 μg/cm^2^ of fibronectin and collagen (Sigma-Aldrich) over-night at 37°C 5% CO_2_. The next day, 300 μl of endothelial cell growth medium were added. Cells were fixed in 4% formaldehyde and then stained for ITGA2b and mCLCA1 with DAPI nuclear counterstain. Images were acquired on a Microscope Zeiss Axio Observer Z1 Confocal LSM780 (Carl Zeiss) with the Carl Zeiss proprietary software Zen and on a spinning disk inverted microscope (Carl Zeiss) with a confocal head Yokogawa CSU and a Metamorph software (Metamorph). Analysis of all microscopic images was done using the open source imageJ-based Fiji distribution.

### Immunofluorescence

Organs were embedded in Tissue-Tek O.C.T Compound (Electron Microscopy Science) and frozen in liquid nitrogen. Six to 8 μm sections were cut, fixed in cold acetone and then blocked with 2% BSA. The following antibodies were used: mCLCA1 (hamster mAb 10.1.1), Lyve-1-A488 (ALY7, eBioscience), ITGA2b-APC (MWReg30, Biolegend), Prox-1 (polyclonal goat, R&D Systems), GPIbß (RAM1-A645), MAdCAM-1 (MECA-367, BD-Pharmingen), fibronectin (Rabbit polyclonal, Patricia Simon-Assmann, UMR-S 1109 INSERM, Strasbourg), RANKL (IK22.5, Rat IgG2a, [22]), goat anti-rabbit-A488 (Molecular Probes), goat anti-hamster-A488/A546 (Molecular Probes), donkey anti-rat-Cy3 (Jackson) or streptavidin A546 or A647 (Molecular Probes). Sections were mounted using DAKO mounting medium (Dako, Hamburg, Germany). Images were acquired and treated as noted above.

### Transmission Electron Microscopy

ITGA2b^+^ sorted LECs were fixed in 2.5% glutaraldehyde for 1 h and 200 μl blue AccuDrops^R^ beads (BD Biosciences) were added to facilitate cell pellet visualization during the subsequence sample preparation as described [23]. Cells were visualized on a CM120 microscope with biotwin lens configuration operating at 120 kV (FEI, Eindhoven, The Netherlands)

### Quantitative Reverse Transcription Coupled Polymerase Chain Reaction (qRT-PCR)

RNA from total LNs and from sorted LECs were extracted with RNeasy kits (Qiagen) and cDNA was synthesized with Maxima First Strand cDNA Synthesis Kit (Thermo Scientific) and Improm-II (Promega) using oligo(dT)15 primers. RT-PCR was performed using Luminaris color HiGreen qPCR Master Mix (Thermo Scientific) using the following primers to amplify ITGA2b: Forward 5’-ATTCCTGTTTAGGACGTTTGGG and Reverse: 5’-TCTTGACTTGCGTTTAGGGC [24] with the housekeeping gene coding for GAPDH (Forward 5’-TGACGTGCCGCCTGGAGAAA and Reverse 5’-AGTGTAGCCCAAGATGCCCTTCAG). Quantitative RT-PCR was run on a Bio-Rad CFX96 thermal cycler, and threshold values (Ct) of the target gene were normalized to GAPDH (ΔCt = CtITGA2b –CtGAPDH). The relative expression was calculated for each sample versus the mean of total 5 day LN ΔCt: ΔΔCt = ΔCtsample– ΔCttotal LN at 5 days; and relative quantification was performed as 2-ΔΔCt.

### Immunization

Six-week-old mice were injected in both posterior limbs with 70 μg of chicken ovalbumin, 600 μg aluminium hydroxide (Sigma-Aldrich) and 6x10^8^ heat inactivated *B*. *pertussis* ml^-1^. A boost was administrated 2 weeks later. Inguinal and popliteal LN were sampled 4 days later.

### Imiquimod Treatment

Adult mice were anesthetized by intraperitoneal injection of ketamine and xylazine (100 μg/g body weight and 10 μg/g body weight, respectively). Back skin or ear skin received 12.5 μg/g (0.1mg/kg body weight) of Toll-like receptor (TLR)-7 agonist imiquimod (Aldara), diluted in neutral cream (Diprobase) [25]. Back skin hair was trimmed before hair removal with cold wax (Klorane, France). The animals were sacrificed 12h after.

### RANKL and Lymphotoxin β Receptor Blockage

The neutralizing anti-RANKL mAb IK22-5 [22] or the rat IgG2a isotype control (BioXell) were administrated s.c. into 6-week old C57BL/6 mice every 3 days (50 μg/ mouse in sterile saline) for two weeks and for 3 consecutive days for the third week. The lymphotoxin β receptor -Ig fusion protein or mIgG1 isotype control (20μg/mouse in sterile saline) were administrated s.c. into 6-week old mice every 3 days for four weeks.

### Statistical Analysis

An unpaired two-tailed Student *t*-test and ANOVA with the Bonferroni method were used to determine statistically significant differences. The p values <0.05 were considered statistically significant. GraphPad Prism version 5 for Windows (GraphPad software) was used for the analysis.

## Results

### LN LECs Express ITGA2b

**Fig 1:**
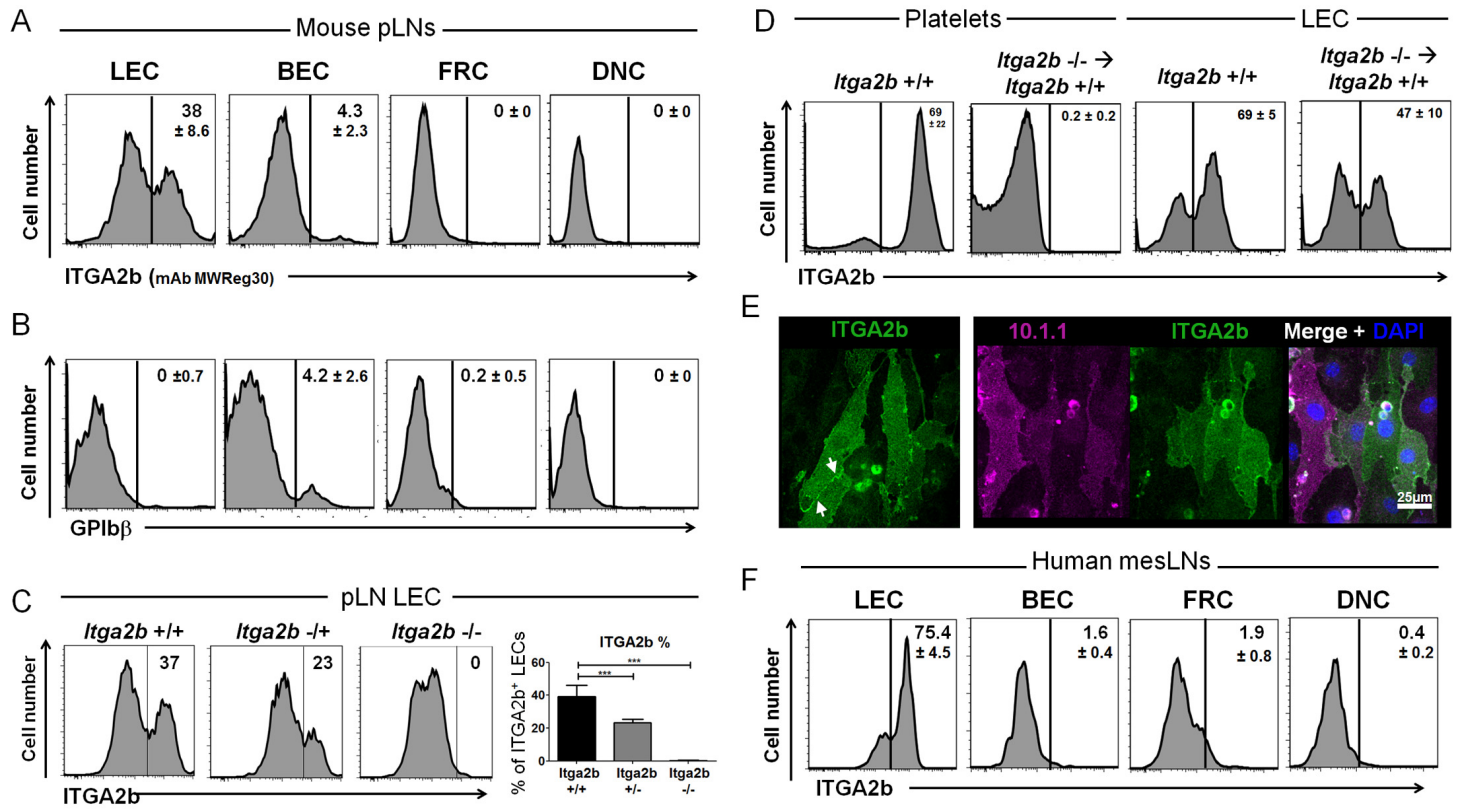
LN LECs express ITGA2b. (A) Flow cytometry histograms display ITGA2b expression by peripheral (p)LN stromal subsets, lymphatic endothelial cells (LEC), blood endothelial cells (BEC), fibroblastic reticular cells (FRC) and the pericyte-containing gp38^-^CD31^-^ double negative cells (DNC). Peripheral LNs are inguinal, brachial and axial LNs. The percentage ±SD (n = 13) of cells labelled by the mAb is indicated. (B) Histograms of the four stromal cell types incubated with a mAb specific for platelet-restricted GPIBβ. The percentage ±SD (n = 8) of cells labelled by the antibody is indicated. (C) Histograms show ITGA2b expression by LECs of WT mice, but reduced and no expression by LECs isolated from mice heterozygous or homozygous for *Itga2b* genetic deletion. The graph depicts the mean ± SD (n = 9) percentage of ITGA2b^+^ LECs in WT controls and in mice heterozygous or homozygous for the *Itga2b* genetic deletion. (D) Histograms of ITGA2b expression by platelets and LECs in control mice and in mice after adoptive transfer of *Itga2b*^*-/-*^ bone marrow (n = 8 for adoptive bone marrow transfer, n = 4 for WT mice). The percentage ±SD of ITGA2b^+^ cells is indicted. (E) Confocal fluorescence microscopy images of cell-sorted LECs in culture showing mCLCA1 (mAb 10.1.1) (magenta) and ITGA2b (green) expression. (F) Flow cytometry histograms ±SD (n = 2) display ITGA2b expression within the four stromal subsets of human embryonic mesenteric LN. ***p<0.001.

Microarray gene expression analysis of murine LN stromal cells had revealed transcription of *Itga2b* by LECs but by no other stromal or hematopoietic cells [26]. To study ITGA2b expression by LECs, we prepared stromal cells from peripheral LNs following the same procedure as used in the microarray study. LECs (gp38^+^CD31^+^), fibroblastic reticular cells (FRCs, gp38^+^CD31^-^), blood endothelial cells (BECs, gp38^-^CD31^+^), and pericyte-containing double-negative cells (DNCs, gp38^-^CD31^-^) were identified in the cell suspension **(panel A in S1 Fig**). ITGA2b-specific mAbs (MWReg30 and RAM-2), validated on platelets (**panel B in S1 Fig**), recognized a major subset of LECs and a minor subset of BECs (**Fig 1A and panel A in S1 Fig**). To verify whether the labelling was due to platelets bound to the cells, we exposed them to an antibody specific for glycoprotein subunit GPIbβ (CD42c) that is exclusively carried by megakaryocytes and platelets [20] (**panel B in S1 Fig**). The antibody recognized the minor fraction of BECs but did not interact with LECs (**Fig 1B**). To further assure that LECs were platelet-free, ITGA2b^+^ LECs were FACS sorted and examined for the adherence of platelets by electron microscopy, however none were found (**panel C in S1 Fig**). To confirm ITGA2b expression, LECs were prepared from mice deficient for ITGA2b [15]. As shown in **Fig 1C**, LECs from heterozygous mice expressed reduced levels of the integrin and LECs from mice with the homozygous deletion fully lacked ITGA2b. We next irradiated WT C57BL/6 mice and adoptively transferred bone marrow from *Itga2b*^*-/-*^ mice. Six weeks later, circulating platelets were devoid of ITGA2b but LECs still expressed the integrin (**Fig 1D** and **panel A in S2 Fig**). Also the staining for platelets on LN cross-sections did not reveal any presence, whereas platelets were found in the spleen red pulp (**panel B in S2 Fig**). We tested whether the integrin could be detected on FACS-sorted LECs grown in culture. The cells were labelled for ITGA2b and the pan-LEC marker mCLCA1 (recognized by mAb 10.1.1, [27,28]). LECs expressing ITGA2b could be seen, which was found distributed throughout the cell and occasionally concentrated at cell-cell junctions (see arrows) (**Fig 1E**). Finally, to extend this finding to man, human embryonic mesenteric LNs were processed in a similar fashion to obtain the four stromal subsets (**panel C in S2 Fig**). An ITGA2b-reactive mAb that recognized platelets from healthy donors but not from an ITGA2b-deficient Glanzmann donor (**panel D in S2 Fig**) labelled LECs but not the other stromal subsets (**Fig 1F**). Taken together, these finding demonstrated that LN LECs, but not other stromal cells, express the ITGA2b integrin.

### ITGA2B Is Restricted to LN LEC Subsets

**Fig 2:**
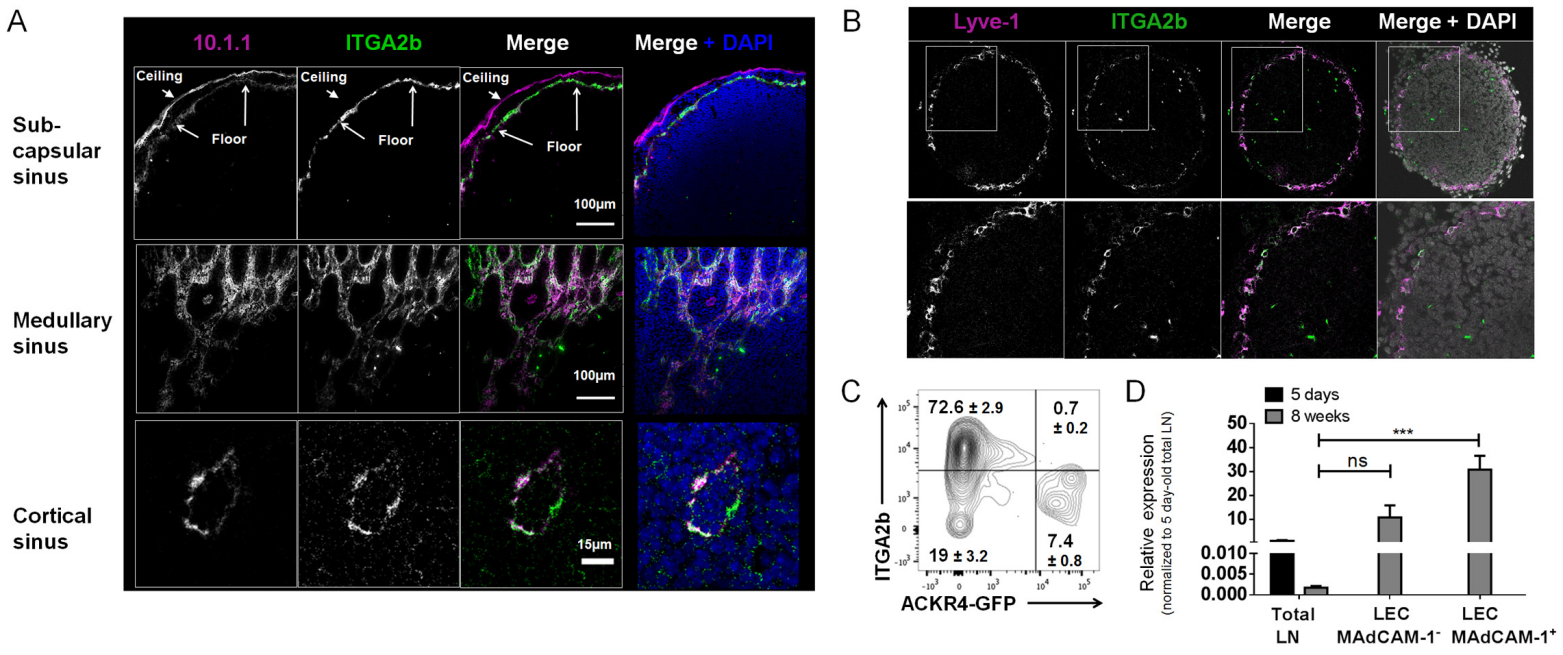
ITGA2b is heterogeneously expressed in the adult and embryonic LN. (A) Confocal microscopy images of an adult inguinal LN probed for ITGA2b together with LEC marker mCLCA1 (mAb 10.1.1) in the subcapsular, the medullary and the cortical sinus. Scale bars are indicated. (B) Confocal microscopy images of an embryonic (E18.5) inguinal LN of ITGA2b and LEC marker Lyve-1. Higher magnification of boxed area is shown below. (C) Flow cytometry counterplot of ITGA2b versus ACKR4 expression by LN LECs of ACKR4-GFP transgenic mice. The percentage ±SD (n = 3) of positive cells is indicated. (D) Mean ± SEM *Itga2b* mRNA expression of total LN from mice aged 5 days and 8 weeks, and of MAdCAM-1^+^ and MAdCAM-1^-^ cell-sorted LECs from mice aged 8 weeks. Statistical analysis: ***p<0.001, ns = non significant by one way Anova with the Bonferroni method.

Because only a proportion of LN LECs expressed ITGA2b, we next wished to localize the ITGA2b^+^ LECs in the mouse LN. ITGA2b immunofluorescence on cross-sections together with LEC marker mCLCA1 (10.1.1 mAb) revealed that LECs of the medullary and the cortical area expressed ITGA2b in a heterogenous manner (**Fig 2A**). Remarkably, the subcapsular sinus ceiling LECs were totally devoid of ITGA2, while its floor counterpart uniformly expressed the integrin. We repeated the immunofluorescence with the lymphatic vessel endothelial hyaluronan receptor (Lyve)-1 and observed again a restricted expression of ITGA2b to a subset of LECs (**panel A in S3 Fig**). Colabelling for Prox-1, the most specific LEC marker, confirmed LEC-restricted ITGA2b staining (**panel B in S3 Fig**). At E18.5, the subcapsular sinus of the embryonic inguinal LNs were not yet formed and was constituted of a single layer of Lyve-1^+^ LECs expressing the integrin to different extents (**Fig 2B**). To verify its exclusion from the subcapsular sinus ceiling-lining cells, LECs of adult mice that express GFP exclusively in this subset [2] were labelled for ITGA2b. Indeed, the ACKR4-GFP^+^ LECs lacked the integrin (**Fig 2C**). To verify ITGA2b expression in the floor-lining cells, we sorted LECs based on gp38/CD31 and MAdCAM-1 expression for qRT-PCR analysis. MAdCAM-1 is uniformly carried by the floor-lining subcapsular sinus LECs but not the ceiling counterpart **(panel A in S4 Fig**) and colabelling showed that all MAdCAM-1^+^ cells express ITGA2b (**panel B in S4 Fig**). MRCs identified by RANKL expression localize close to the LECs but do not carry the integrin (**panel C in S4 Fig**). In whole adult LN, *Itga2b* mRNA was barely detectable, however sorted MAdCAM-1^+^ and MAdCAM-1^-^ cells clearly expressed the message (**Fig 2D**). Sorted LN BECs were devoid of the mRNA (data not shown). This supports its restricted expression in LECs, and in particular the floor-lining MAdCAM-1^+^ LECs. To determine whether ITGA2b is expressed by non-LN LECs, skin was processed into a cell suspension and LECs were identified as CD45^-^F4/80^-^gp38^+^CD31^+^ cells. However, we saw no ITGA2b expression by FACS or qRT-PCR, neither in LECs from resting skin, after activation with imiquimod [25] or from RANK-transgenic mice overexpressing RANKL from hair follicles [14,29] (**panel D in S4 Fig** and data not shown). Taken together, the data show that ITGA2b is restricted to subsets of LN LECs.

### LEC ITGA2b Is Not Required for Residence in Fibronectin-Rich Environments

**Fig 3:**
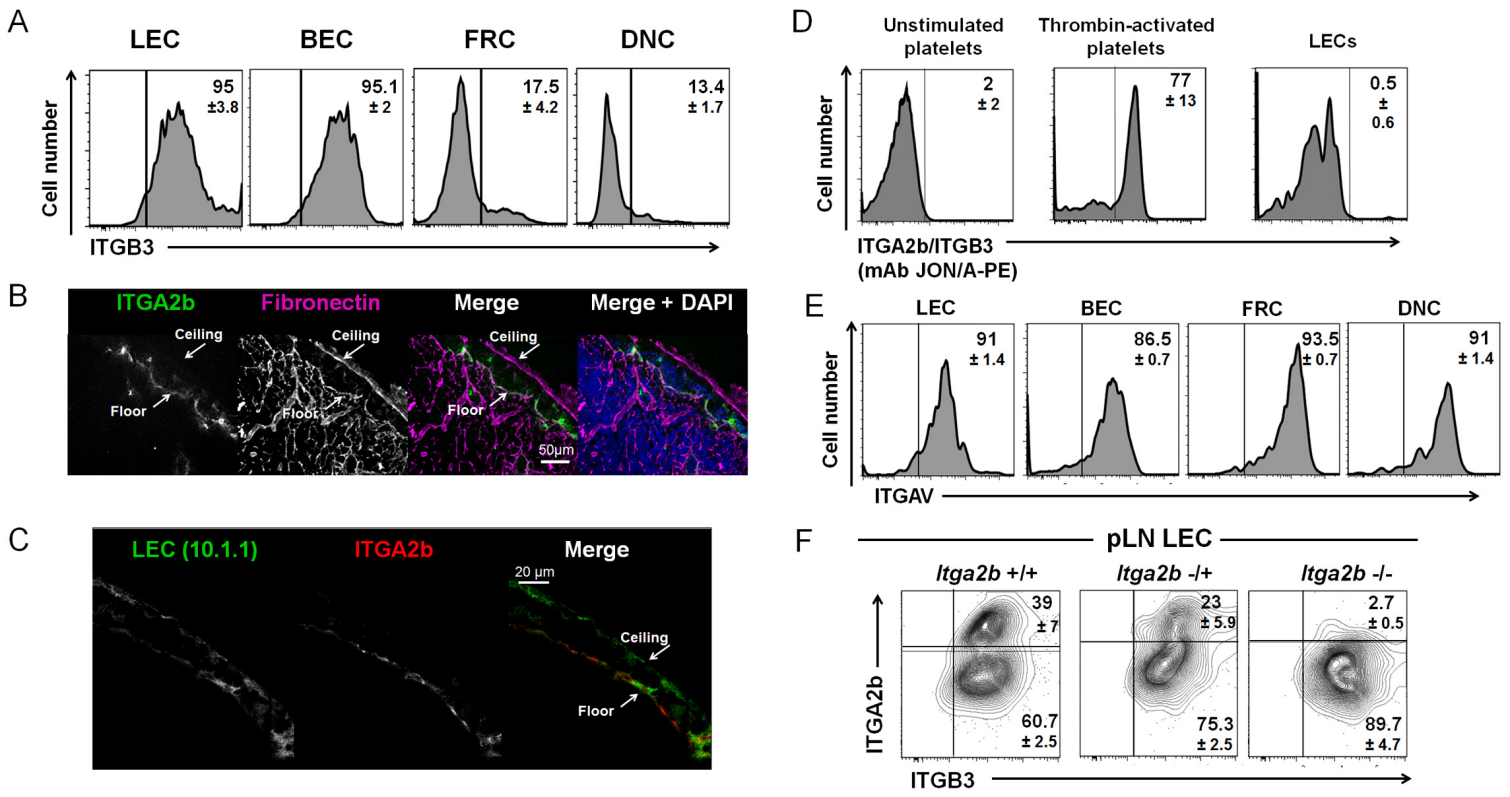
LEC ITGA2b is not required for residence in fibronectin-rich environments. (A) Flow cytometry histograms show the percentage ±SD (n = 4) of LECs expressing ITGB3 by the four stromal subsets. (B) Confocal microscopic images of a LN subcapsular area labelled for fibronectin (magenta) and ITGA2b (green). Nuclear coloration was with DAPI. (C) Confocal high resolution microscopy of the subcapsular area labelled for ITGA2b and mCLCA1 (mAb 10.1.1). (D) Histograms show recognition of the active conformation of the ITGA2b/ITGB3 complex on activated platelets but not on LECs or unstimulated platelets by the PE-conjugated JON/A mAb. (E) Histograms show ITGAV expression ±SD (n = 3) on the stromal subsets. (F) Flow cytometry counterplots of LECs probed for expression of ITGA2b and ITGB3 in *Itga2b*^+/+^, *Itga2b*^+/-^ and *Itga2b*^-/-^ mice (n = 3).

To explore the function of ITGA2b for LECs, we first asked whether ITGA2b could heterodimerize with ITGB3 to interact with ligands, such as fibronectin. Indeed, ITGB3 was uniformly expressed by LECs and BECs, while there was little of the β-chain found on FRCs and DNCs (**Fig 3A**). We next assessed whether there was a correlation between ITGA2b expression and residence in a fibronectin-containing environment. To this end, we stained the LN subcapsular area that contained the ITGA2b^+^ floor-lining and the ITGA2b^-^ ceiling-lining LECs for fibronectin. It was apparent that this extracellular matrix component was present in both sites, demonstrating that the absence of ITGA2b does not prevent LECs to take up position in the fibronectin-containing ceiling (**Fig 3B**). Further, the positioning of the integrin relative to the sinus luminal or abluminal side was investigated and no preferential location was found (**Fig 3C**). To explore whether a functional ITGA2b/ITGB3 complex was indeed formed, LECs and, as controls unstimulated and thrombin-activated platelets, were incubated with the phycoerythrin (PE)-conjugated JON/A mAb, which recognizes only the ITGA2b/ITGB3 complex in its activated state, as found on platelets [30,31]. While agonist stimulated platelets were labelled with this mAb, LECs were not recognized (**Fig 3D**). This suggests that if an ITGA2B/ITGB3 heterodimer is formed on LECs, it is not in a configuration recognized by PE-JON/A and may present a low affinity for its ligands. We therefore asked whether ITGA2b could be substituted by ITGAV to pair with ITGB3. LECs, alike all other stromal cells, expressed ITGAV, suggesting that they could anchor to matrix proteins through ITGAV/ITGB3 (**Fig 3E**). In platelets (**panel B in S1 Fig**) but not in embryonic hematopoietic stem cells or mast cells [24,32], ITGA2b is required to translocate ITGB3 to the cell surface [15,33], raising the question of whether, in the absence of ITGA2b, ITGB3 would be available to heterodimerize with ITGAV on the cell surface. To assess this issue, we labelled LECs from mice deficient for ITGA2 and observed that cell surface expression of the ITGB3 was maintained in *Itga2b*^-/-^ mice (**Fig 3F**). Taken together, ITGA2b is not required for residence of the ceiling LECs in its fibronectin-containing environment, most probably by the formation of an ITGAV/ITGB3 complex that binds with high affinity to this matrix protein.

### The ITGA2b^+^ Subset Is Sensitive to Activation by RANKL and Lymphotoxin

**Fig 4:**
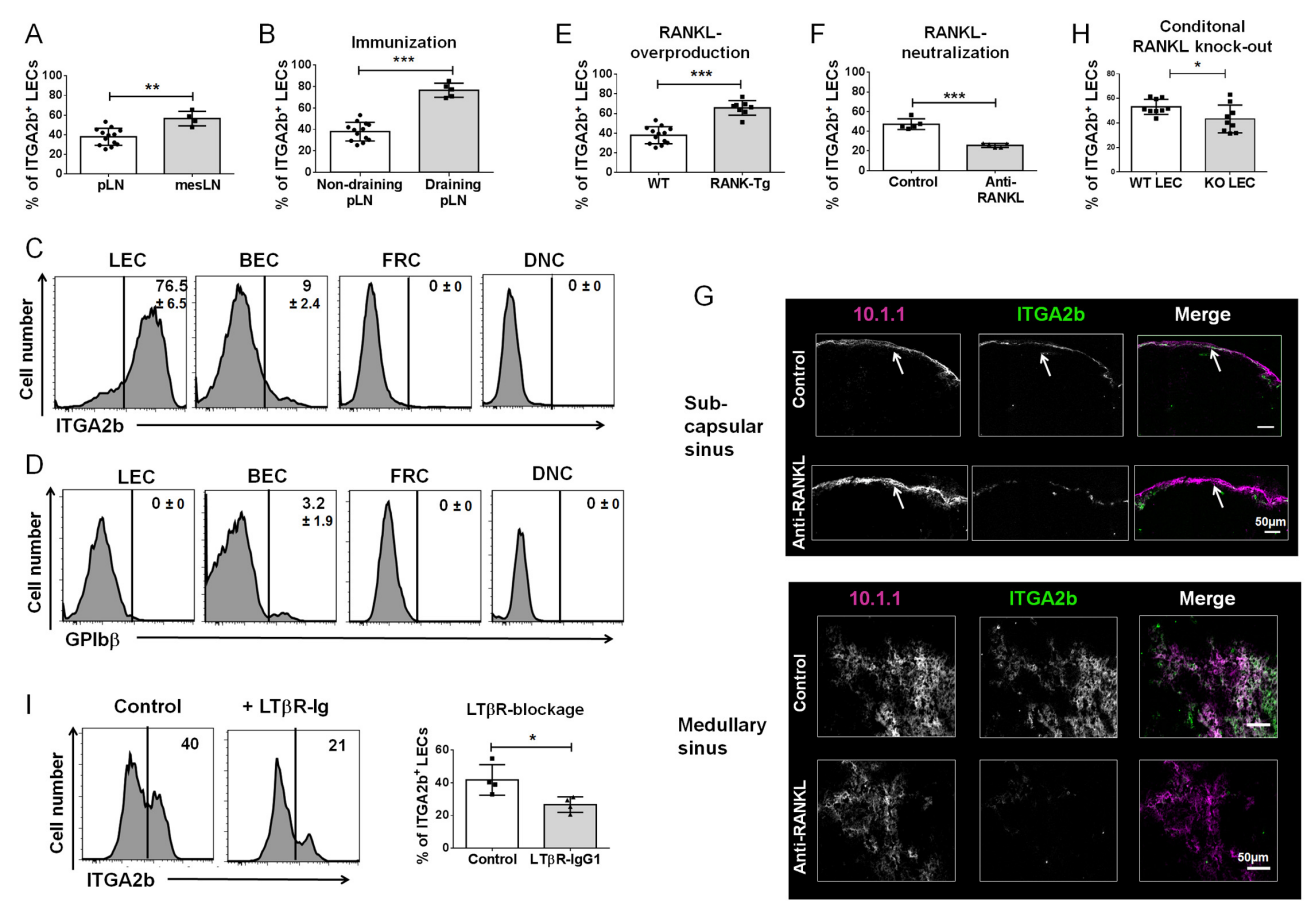
ITGA2b^+^ LECs are responsive to RANKL and lymphotoxin activation. (A) Mean ± SD (n = 6) percentage of ITGA2b^+^ LECs of peripheral (p)LNs (inguinal, axial and brachial) versus mesenteric (mes) LNs. (B) Mice were immunized with heat-inactivated *B*. *pertussis* and LEC ITGA2b expression of draining and non-draining LNs was compared. The graph shows the mean ± SD (n = 6) percentage of ITGA2b^+^ LECs, revealing increased ITGA2b proportions in response to immunization. (C) Flow cytometry histograms display representative ITGA2b expression ±SD (n = 6) by stromal subsets of inguinal and popliteal LNs draining the immunization site. (D) Histograms show reactivity to anti-GPIBβ labelling of stromal subsets of inguinal and popliteal LNs draining the immunization site. The percentage ±SD (n = 6) of cells labelled by the antibody is indicated. (E) The increase in the proportion of ITGA2b^+^ pLN LECs from RANK-Tg mice (overproducing soluble RANKL in the skin) compared with LECs of WT controls is shown as mean ± SD (n = 8). (F) Graph shows reduction in the percentage of ITGA2b^+^ LECs upon RANKL neutralization (mean ± SD, n = 5). (G) Confocal microscopy imaging in inguinal LN subcapsular and medullary sinuses of ITGA2b expression (green) by LECs (10.1.1, magenta) after RANKL-neutralization or after administration of isotype-control antibody. (H) Mean ± SD (n = 9) percentage of ITGA2b^+^ LECs from mice with conditional deficiency of RANKL in marginal reticular cells (KO) versus WT littermate controls. (I) Histograms of ITGA2b expression of LECs from mice treated with LTβR-Ig or IgG1 control. The graph depicts the mean ± SD (n = 3) percentage of ITGA2b^+^ LECs. *p<0.5, **p < 0.01, ***p < 0.001.

We noted that in comparison to peripheral LNs, the proportion of ITGA2b^+^ LECs was higher in mesenteric LNs (**Fig 4A**). Because mesenteric LNs are stimulated by the intestinal microflora, this evoked the possibility that the heterogenous LN ITGA2b expression reflects differences in cell activation. To test this hypothesis, we administered heat-inactivated *Bordetella pertussis* subcutaneously, and after a secondary immunization, compared ITGA2b expression in draining and non-draining LNs. The proportion of ITGA2b^+^ LECs was markedly increased in response to immunization (**Fig 4B**), while the other stromal cells remained devoid of the integrin (**Fig 4C**). The upregulation was not due to platelet adherence to LECs because there was no recognition of LECs by the GPIbβ-specific mAb (**Fig 4D)**. We also tested whether the innate immune stimulus imiquimod (TLR7 ligand) resulted in a similar upregulation. LECs from auricular LNs draining imiquimod or mock-treated ears were analyzed, however, the proportion of ITGA2b^+^ LECs did not rise after application of the TLR-7 ligand (**panel A in S5 Fig**). We have previously observed that RANKL activates LN LECs in a transgenic model of cutaneous RANKL overproduction [14]. Therefore, we determined in these mice whether RANKL affected ITGA2b levels and indeed found that ITGA2b expression was positively regulated by this TNF-family member (**Fig 4E**). We addressed the question of whether integrin upregulation was due to its externalization to the cell surface. In WT controls, immunolabelling of permeabilized cells revealed a stronger signal compared with the cell surface, however, the signal was identical in the LECs isolated from the transgenic mice (**panel B in S5 Fig**). This suggests that the increased expression of ITGA2b by RANKL stimulation likely involves its translocation from the cytoplasm to the cell membrane. We tested whether the upregulation was accompanied by a rise in transcriptional activity. In comparison with WT controls, there was no major increase in mRNA synthesis in the LEC subsets isolated from the mutant mice (**panel C in S5 Fig)**. However, because the proportion of MAdCAM-1^+^ LECs greatly augmented in the transgenic mice (**panel C in S5 Fig**), the increase in ITGA2b expression in these mice is principally the result of an expansion of the MAdCAM-1^+^ subset that naturally expresses more ITGA2b. We next determined if neutralizing RANKL in WT mice led to a downregulation of ITGA2b. Administration of RANKL-blocking mAb caused a significant decrease in ITG2b expression by LECs in comparison to isotype injected controls (**Fig 4F**). Immunofluorescence on sections confirmed the strong decline of ITGA2b in subcapsular and medullary sinuses (**Fig 4G**). Because in the LN, RANKL is principally produced by the marginal reticular cells (MRCs) [13], this raised the possibility that MRC RANKL activates LECs resulting in ITGA2b expression. To address this question, we generated mice conditionally deficient for RANKL in MRCs by crossing *Ccl19-cre* mice [16] with *Rankl*^*f/f*^ mice [17]. These mice were devoid of RANKL expression by MRCs (**panel D in S5 Fig**). Analysis of RANKL^ΔCCL19^ mice showed that the disappearance of MRC RANKL significantly compromised ITGA2b expression (**Fig 4H**), supporting a role of RANKL in LEC activation. However, because there was not a complete loss of ITGA2b other factors could contribute to LEC activation. Indeed, approximately 15% of LECs were double positive for RANK and ITGA2b, suggesting that a proportion of LECs reacts to other stimulatory factors (**panel E in S5 Fig**). On the other hand, cutaneous LECs that do not respond to RANKL do not carry any RANK (**panel F in S5 Fig**). In light of similar activities of RANKL and lymphotoxin αβ (LT) [10,34] and the expression of the LTβ receptor (R) by LECs [35], we asked whether also LT regulated ITGA2b expression. Therefore, mice were treated with soluble LTβR-Ig to inhibit LTβR signaling [36]. We found that this treatment likewise reduced ITGA2b (**Fig 4I**). Taken together, ITGA2b is a novel marker for subsets of LN LECs that respond to activation by TNF-family members RANKL and LT.

## Discussion

In this study we show that ITGA2b is expressed by a subset of LN LECs in the subcapsular, cortical and medullary sinus. This subset is also marked by MAdCAM-1. The ITGA2b^+^ population is sensitive to stimulation by the TNFSF members RANKL and LT.

ITGA2b is known to be carried by megakaryocytes and platelets as well as by hematopoietic stem and progenitor cells in the embryo and the adult [7–9,37]. Here we show for the first time that LN LECs also carry this integrin. A number of studies had analyzed ITGA2b expression using different experimental approaches, including a genetic reporter system to mark ITGA2b-expressing cells by β-galactosidase [7–9]. However, these reports, which investigated whole embryos, the embryonic aorta-gonad-mesonephros region, spleen, thymus and bone marrow, did not analyze LNs. Although platelets interact with endothelial cells in the embryo during separation of blood and lymphatic systems [4], the following observations exclude the possibility that ITGA2b expression by LEC is the result of platelet contamination: (i) the platelet glycoprotein subunit Iβ was not detected on LECs, (ii) electron microscopy did not reveal platelets adhering to the cells, (iii) ITGA2b-deficient platelets lacked surface ITGB3, yet the β-chain was expressed by LECs of *Itga2b*^*-/-*^ mice, (iv) after adoptive transfer of *Itga2b*^*-/-*^ bone marrow resulting in the repopulation of ITGA2b^-^ platelets, the integrin was still expressed by LECs and (vi) *Itga2b* mRNA was amplified from sorted LECs. The related BECs were devoid of the integrin, irrespective of the site of residence or the presence of stimulatory signals. This is supported by an early report noting the absence of ITGA2b in the blood endothelial cell line bEnd3 [38].

LN LECs and BECs uniformly expressed ITGB3 and ITGAV, while only a subset of LECs also carried ITGA2b. Both α-chains pair with ITGB3 and recognize similar matrix proteins, such as fibronectin, fibrinogen, von Willebrand factor and vitronectin, which raises the question of the necessity of the ITGA2b chain. This is in contrast to platelets that predominantly express ITGA2b to ensure platelet aggregation. Indeed, although ITGA2b was expressed by LECs in embryonic LNs, its absence had no discernible impact on LN development. In addition, those LECs that naturally lack ITGA2b are still capable of taking up residence in the fibronectin-rich subcapsular sinus. Further, the absence of polarization towards the luminal or the abluminal side does not point to a predominant function in cell-cell interaction or cell-extracellular matrix contact. It is likely that LECs rendered genetically deficient for ITGA2b function normally, since the migration of tissue-derived dendritic cells to the LN cortex of ITGA2b-deficent mice was unperturbed (data not shown). Although we observed a reduction in the number of B cells, it cannot be excluded that this defect was the result of a loss of ITGA2b from platelets (data not shown). Indeed, a minor defect in LN structuring during development was seen in mice lacking platelet CLEC-2 [39]. Further investigation into the specific role of ITGA2b for LEC function will await the generation of mice with conditional deletion of ITGA2b in these cells. Inside-out signaling of platelets results in a conformation change of ITGA2b/ITGB3 to increase affinity for its ligands. This conformation is detected by the PE-conjugated JON/A mAb. LECs were not recognized by the antibody indicating either that ITGA2b does not pair with ITGB3 or that the complex is not in the same configuration as that found on platelets. On the other hand, to our knowledge, this mAb has only been used successfully on activated platelets and may not be a suitable reagent to probe for the ITAG2b/ITGB3 heterodimer on other cells. It is also noteworthy that although bone marrow-derived mast cells express ITGA2b and ITGB3, no binding to fibrinogen was seen, and, paradoxically, cell adhesion to fibronectin increased in ITGA2b-deficient cells [24]. It should also be noted that the densities of the α and β chains are at least 10-fold higher on platelets owing to their approximately 10-fold smaller size with roughly equal mean fluorescence intensities, resulting in greatly increasing the avidity.

ITGA2b was carried by the subcapsular floor-lining LECs but absent from its ceiling equivalent. Interestingly, this expression pattern was shared with MAdCAM-1. Hence, MAdCAM-1^+^ LECs displayed the highest *Itga2b* transcriptional activity. In addition, there was a heterogenous expression of ITGA2b in the medullary and the cortical sinuses. Skin LECs were devoid of the integrin on protein and mRNA levels in all conditions tested. Difference in tissue versus secondary lymphoid organ LECs is supported by other examples, such as Sphingosine-1-phosphate [3], found expressed by LN LECs, or ITGA9 [40] that is carried exclusively by vessel LECs. In view of its uniform expression by the subcapsular floor-lining LECs, their juxtaposition to the RANKL-expressing MRCs, and the finding that RANKL upregulates MAdCAM-1 expression [14], we reasoned that RANKL may control ITGA2b synthesis. Using overexpression and neutralization / genetic deletion, we showed that RANKL positively regulates the proportion of ITGA2b^+^ LECs. The finding that conditional deficiency of RANKL from MRCs lowers ITGA2b expression to the same extent as RANKL neutralization concords with the idea that MRC RANKL is the main LN RANKL source and identifies a cellular target for the stromal cell-produced RANKL. However, in the absence of definite proof that RANKL activates ITGA2b transcription we cannot completely rule out the possibility that RANKL stimulates the expansion of the ITGA2b^+^ subset. Two elements suggested that RANKL is not the exclusive ITGA2b regulatory factor: (i) RANKL neutralization or genetic deletion do not eliminate its expression and (ii) only a proportion of ITGA2b^+^ LECs express RANK. Lymphotoxin and RANKL share not only biological functions (requirement for secondary lymphoid organ formation), signaling (canonical and non-canonical NF-κB pathways) but also receptor expression by LECs, so that it appeared rational to investigate the impact of LTβR blockage. Indeed, administration of LTβR-Ig also led to reduced ITGA2b expression. It is therefore likely that both RANKL and LT contribute to the expression of this integrin by LECs and that its upregulation in response to immunization is the consequence of stimulatory factors including RANKL produced by primed T cells and LT expressed by activated B and T cells. The finding that imiquimod had no effect on ITGA2b may therefore reflect a failure to stimulate RANKL and LT synthesis. Further work is necessary to determine whether other stimuli such as TNF-α or T and B cell-released cytokines also impact on ITGA2b expression by LN LECs. Beyond the question of its function for LN LECs, the ITGA2b integrin sheds a new light on the heterogeneity of LECs and their response to activation signals.

## Supporting Information

S1 Fig (A) Left: Flow cytometry dot plot profiles displaying the gating strategy for stromal cell identification in CD45/Ter119-depleted LN cell suspensions. Right: Flow cytometry histograms show ITGA2b expression by the four stromal subsets using the RAM-2 mAb. The percentage ±SD (n = 6) of cells labelled by the antibody is indicated. (B) Validation of MWReg30 and RAM.2 (anti-ITGA2b antibodies), RAM.1 (anti-GPIbβ antibody) and 2C9.G2 (anti-ITGB3 antibody) in WT and knock-out animals. Expression of ITGA2b and ITGB3 was seen on platelets from *Itgab2*^+/+^ mice but not on platelets from *Itgab2*^-/-^ mice. GPIbβ was present on platelets of both mice. (C) FACS sorting of Itga2b^+^ LECs. Plots for sorting and post-sort analyses are represented. Sorted cells were then viewed by transmission electron microscopy. Eight representative images of LECs are shown together with an image of platelets with the same magnification.
(TIF)

S2 Fig (A) Microscopy images of a LN of mouse that was lethally irradiated and had received *Itgab2*^-/-^ bone marrow, labelled for ITGA2b (clone MWReg30) and the LEC marker mCLCA1 (mAb 10.1.1). (B) Staining of spleen and LN sections for platelets using the platelet-specific glycoprotein GPIbβ (mAb RAM.1) together with LEC marker mCLCA1 (mAb 10.1.1). RP = red pulp. (C) Flow cytometry dot plot profiles displaying the gating strategy for stromal cell identification from human embryonic mesenteric LN. (D) The histogram displays ITGA2b expression (SDF.2 mAb) on human healthy donor platelets but not on platelets from a patient with Glanzmann’s thrombasthenia. Representative image of over 10 donors.
(TIF)

S3 Fig (A) Confocal microscopy images of an inguinal LN, showing the medullary and the subcapsular sinus LECs labelled with anti-Lyve-1 and anti-ITGA2b mAbs. Images are representative of 2 different experiments. (B) Upper: Images of the medullary region stained for ITGA2b and nuclear Prox-1. Lower: Images of the subcapsular sinus from a LN of a mouse expressing GFP under the control of the Prox-1 promoter.
(TIF)

S4 Fig (A) Confocal microscopy images of a LN subcapsular sinus showing MAdCAM-1 expression by the floor-lining but not the ceiling-lining LECs marked with the 10.1.1 mAb. (B) Images show overlapping staining of subcapsular sinus LECs for mCLCA1 (mAb 10.1.1), MAdCAM-1 (MECA-367) and ITGA2b (MWReg30). The flow cytometry profile shows double labelling of LECs with ITGA2b and MAdCAM-1. (C) Confocal microscopy images of a LN subcapsular sinus labelled for MAdCAM-1 and RANKL. (D) Flow cytometry of mouse skin: upper dot plot panels depict the gating strategy for skin LECs; lower panels show the histograms for ITGA2b expression ± SD (n = 3) of skin LECs from control mice, imiquimod-treated skin and from RANK-transgenic skin (overexpressing RANKL in the hair follicles).
(TIF)

S5 Fig (A) Histograms show ITGA2b expression of auricular LN LECs from mice non-treated or after 2 or 4 day topical application of imiquimod on ears. Graph depicts the levels (geometric mean of fluorescence) of ITGA2b expression (mean ±SD, n = 3–4). (B) Histograms show LEC ITGA2b expression on the cell surface or on the cell surface and in the cytoplasm for WT and RANK-Tg mice. The graphs show the expression levels of the integrin in LECs after cell surface or intracellular/cell surface labelling. The data for WT mice are of 8 mice and for Tg mice are of 4 mice. (C) Graphs show mean ± SEM (n = 6) *Itga2b* mRNA expression of total LN, MAdCAM-1^+^ and MAdCAM-1^-^ LECs from WT and RANK-Tg mice (left) normalized with respect to WT 5 day LNs. Right: Graph shows the mean ± SD (n = 10) percentage of MAdCAM-1^+^ LECs in WT and RANK-Tg mice measured by flow cytometry. (D) Confocal microscopy images of WT and RANKL^ΔCcl19^ inguinal LNs, showing the subcapsular sinus area labelled for mCLCA1 (red) and RANKL (green). The RANKL^ΔCcl19^ LN is devoid of RANKL expression. Representative of 4 mice. (E) Counterplot of LN LECs double stained for ITGA2b and RANK expression. Graph bar (n = 6) shows the percentage of LECs expressing both ITGA2b and RANK. (F) Histograms show RANK expression by LECs and BECs from skin and LNs. The percentage of ITGA2b^+^ cells is indicated. Graph shows their mean ± SD (n = 6) percentages. ns = not significant, *p<0.5, **p < 0.01, ***p < 0.001.
(TIF)
