# Supplementary figures and images for: Integrin-Alpha IIb Identifies Murine Lymph Node Lymphatic Endothelial Cells Responsive to RANKL
Source: PLoS One. 2016 Mar 24;11(3):e0151848. doi: 10.1371/journal.pone.0151848 (PMC4806919; doi:10.1371/journal.pone.0151848)

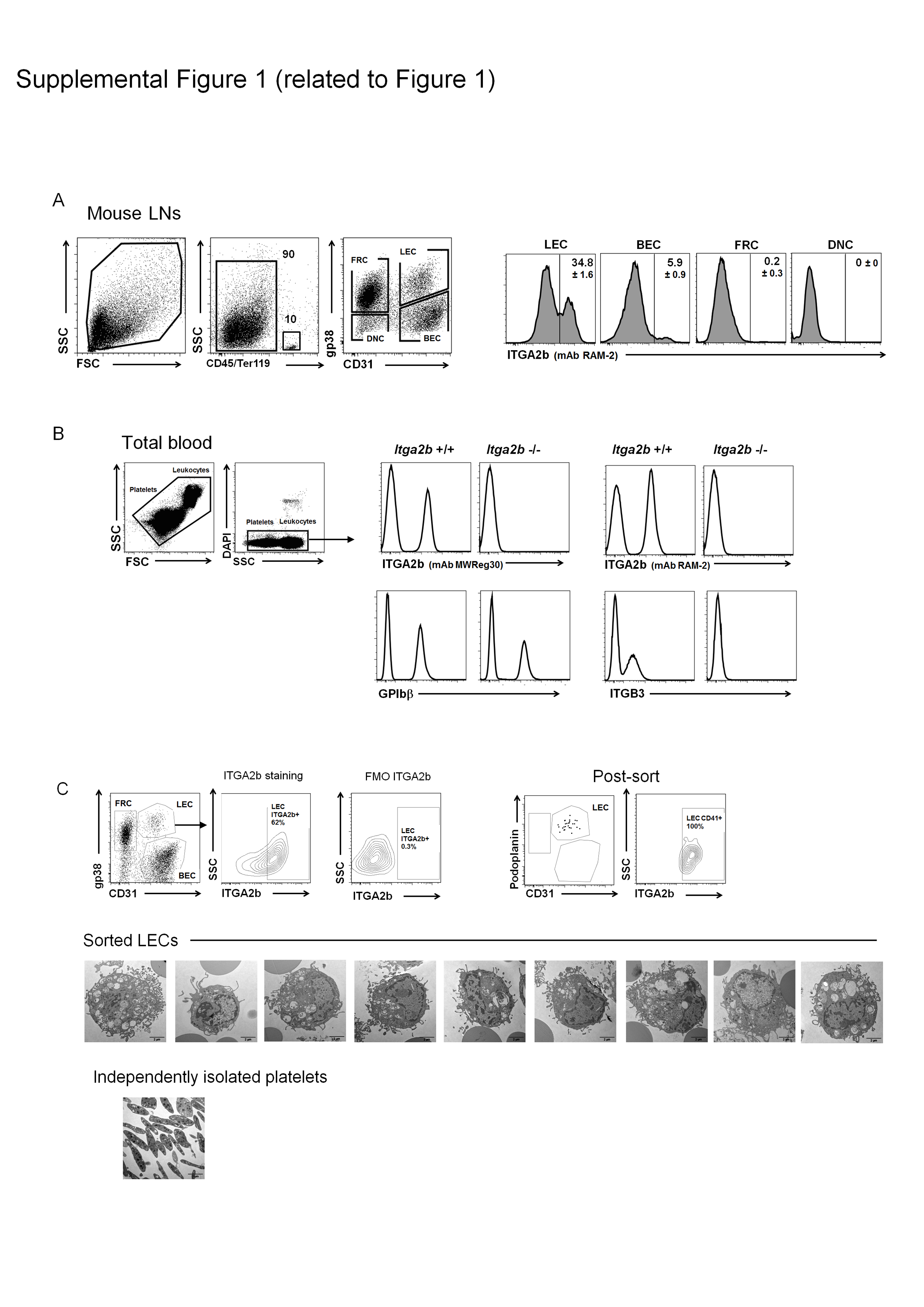

Supplement: S1 Fig — (A) Left: Flow cytometry dot plot profiles displaying the gating strategy for stromal cell identification in CD45/Ter119-depleted LN cell suspensions. Right: Flow cytometry histograms show ITGA2b expression by the four stromal subsets using the RAM-2 mAb. The percentage ±SD (n = 6) of cells labelled by the antibody is indicated. (B) Validation of MWReg30 and RAM.2 (anti-ITGA2b antibodies), RAM.1 (anti-GPIbβ antibody) and 2C9.G2 (anti-ITGB3 antibody) in WT and knock-out animals. Expression of ITGA2b and ITGB3 was seen on platelets from Itgab2+/+ mice but not on platelets from Itgab2-/- mice. GPIbβ was present on platelets of both mice. (C) FACS sorting of Itga2b+ LECs. Plots for sorting and post-sort analyses are represented. Sorted cells were then viewed by transmission electron microscopy. Eight representative images of LECs are shown together with an image of platelets with the same magnification. (TIF) [file pone.0151848.s001.tif]

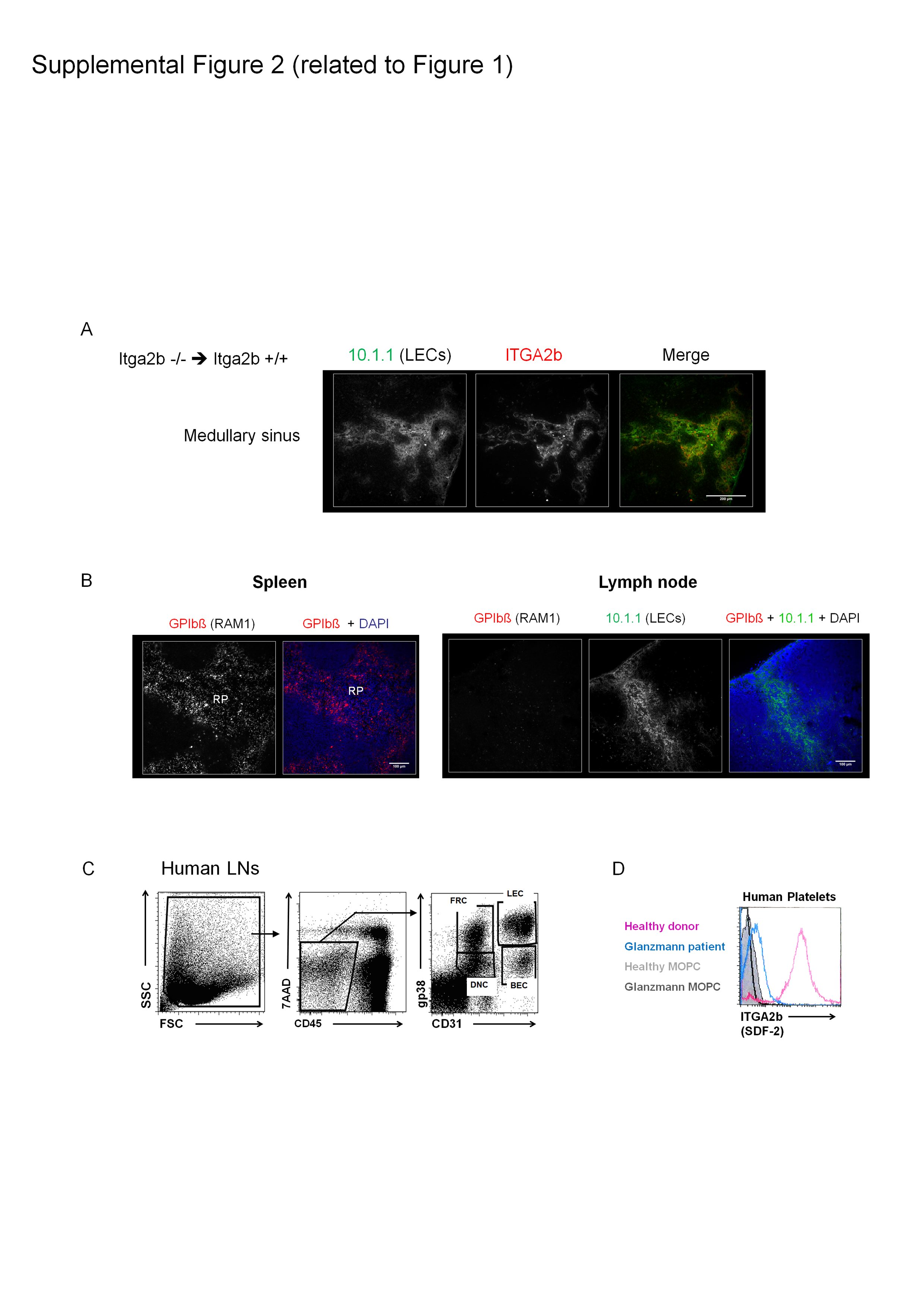

Supplement: S2 Fig — (A) Microscopy images of a LN of mouse that was lethally irradiated and had received Itgab2-/- bone marrow, labelled for ITGA2b (clone MWReg30) and the LEC marker mCLCA1 (mAb 10.1.1). (B) Staining of spleen and LN sections for platelets using the platelet-specific glycoprotein GPIbβ (mAb RAM.1) together with LEC marker mCLCA1 (mAb 10.1.1). RP = red pulp. (C) Flow cytometry dot plot profiles displaying the gating strategy for stromal cell identification from human embryonic mesenteric LN. (D) The histogram displays ITGA2b expression (SDF.2 mAb) on human healthy donor platelets but not on platelets from a patient with Glanzmann’s thrombasthenia. Representative image of over 10 donors. (TIF) [file pone.0151848.s002.tif]

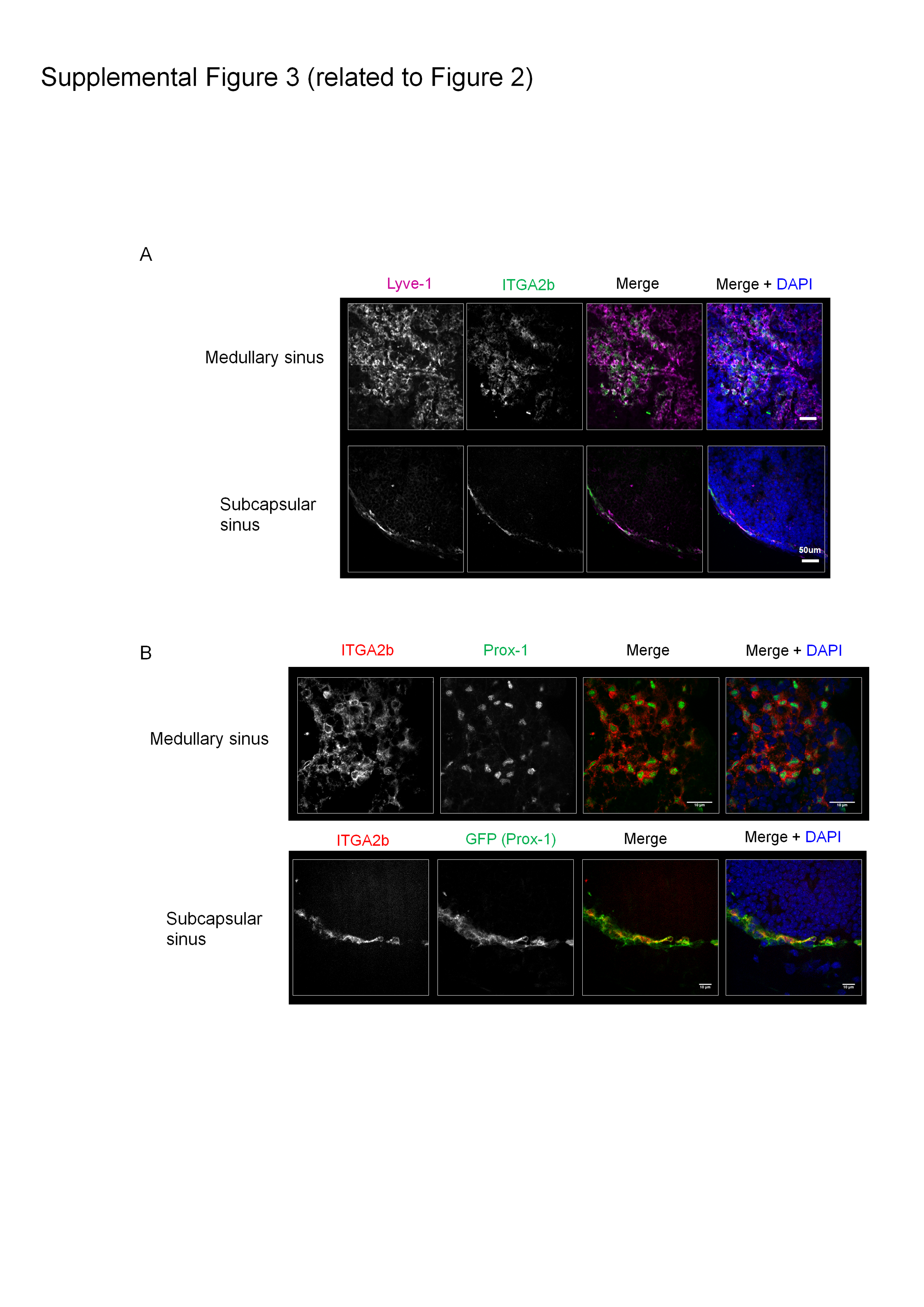

Supplement: S3 Fig — (A) Confocal microscopy images of an inguinal LN, showing the medullary and the subcapsular sinus LECs labelled with anti-Lyve-1 and anti-ITGA2b mAbs. Images are representative of 2 different experiments. (B) Upper: Images of the medullary region stained for ITGA2b and nuclear Prox-1. Lower: Images of the subcapsular sinus from a LN of a mouse expressing GFP under the control of the Prox-1 promoter. (TIF) [file pone.0151848.s003.tif]

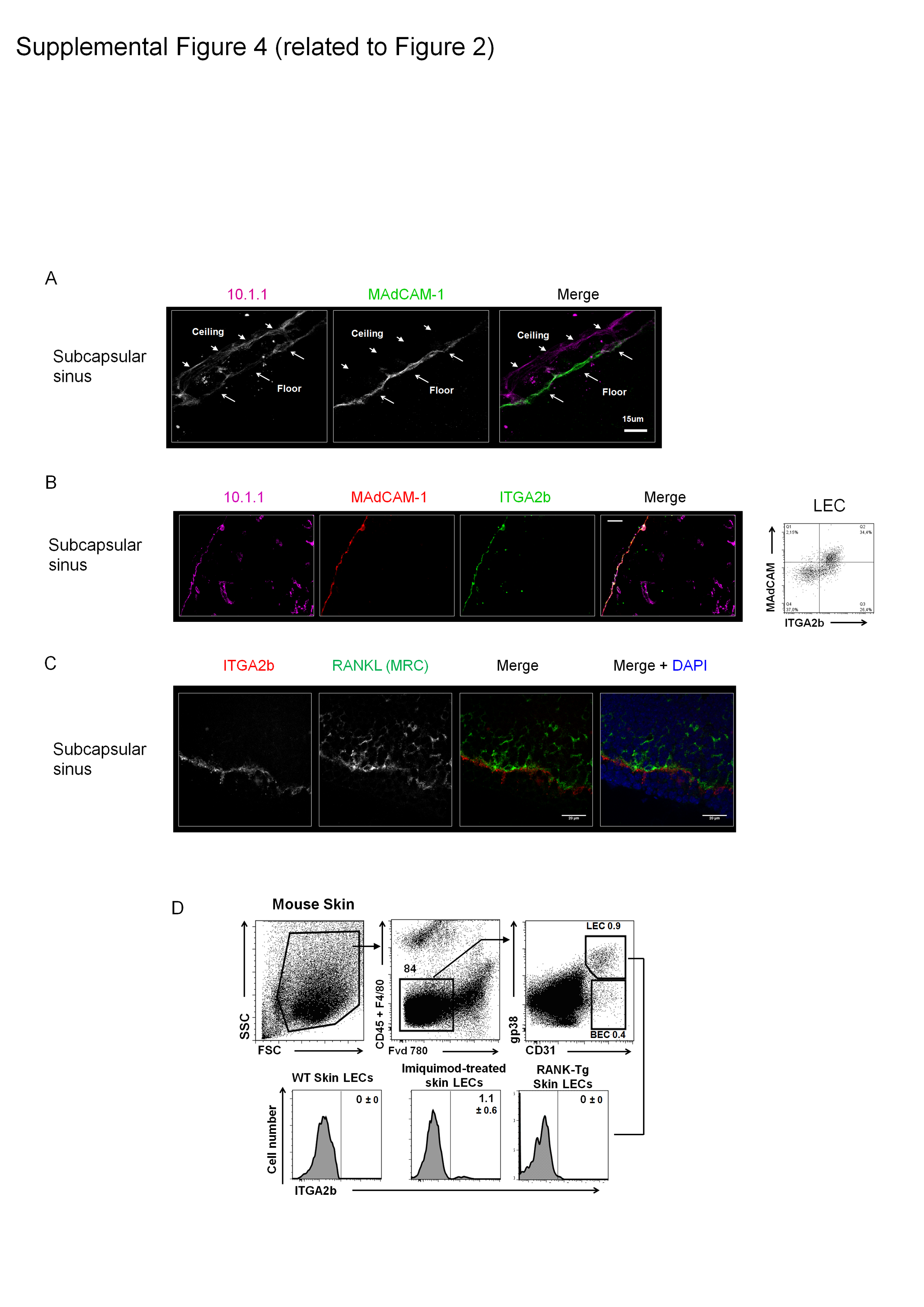

Supplement: S4 Fig — (A) Confocal microscopy images of a LN subcapsular sinus showing MAdCAM-1 expression by the floor-lining but not the ceiling-lining LECs marked with the 10.1.1 mAb. (B) Images show overlapping staining of subcapsular sinus LECs for mCLCA1 (mAb 10.1.1), MAdCAM-1 (MECA-367) and ITGA2b (MWReg30). The flow cytometry profile shows double labelling of LECs with ITGA2b and MAdCAM-1. (C) Confocal microscopy images of a LN subcapsular sinus labelled for MAdCAM-1 and RANKL. (D) Flow cytometry of mouse skin: upper dot plot panels depict the gating strategy for skin LECs; lower panels show the histograms for ITGA2b expression ± SD (n = 3) of skin LECs from control mice, imiquimod-treated skin and from RANK-transgenic skin (overexpressing RANKL in the hair follicles). (TIF) [file pone.0151848.s004.tif]

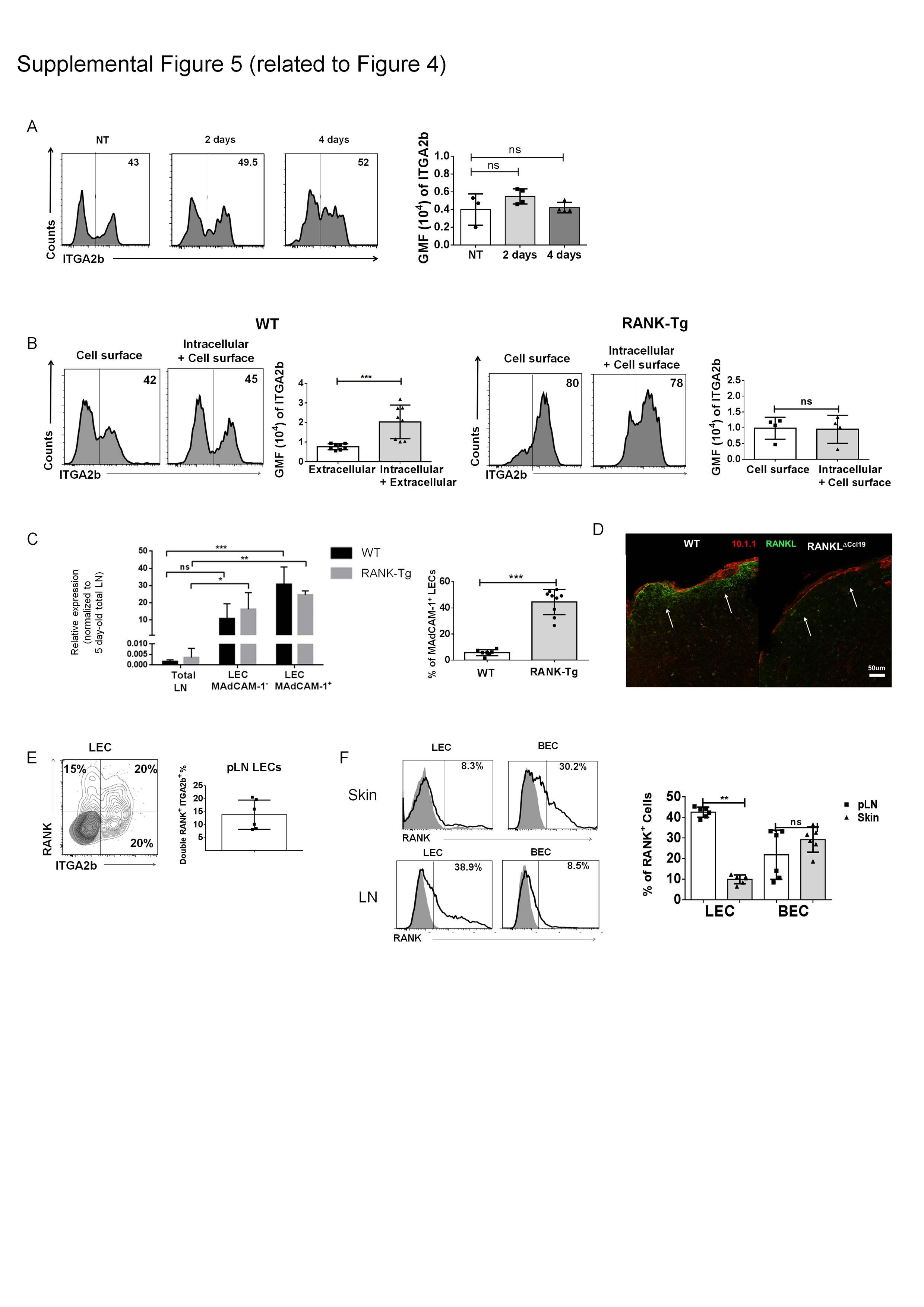

Supplement: S5 Fig — (A) Histograms show ITGA2b expression of auricular LN LECs from mice non-treated or after 2 or 4 day topical application of imiquimod on ears. Graph depicts the levels (geometric mean of fluorescence) of ITGA2b expression (mean ±SD, n = 3–4). (B) Histograms show LEC ITGA2b expression on the cell surface or on the cell surface and in the cytoplasm for WT and RANK-Tg mice. The graphs show the expression levels of the integrin in LECs after cell surface or intracellular/cell surface labelling. The data for WT mice are of 8 mice and for Tg mice are of 4 mice. (C) Graphs show mean ± SEM (n = 6) Itga2b mRNA expression of total LN, MAdCAM-1+ and MAdCAM-1- LECs from WT and RANK-Tg mice (left) normalized with respect to WT 5 day LNs. Right: Graph shows the mean ± SD (n = 10) percentage of MAdCAM-1+ LECs in WT and RANK-Tg mice measured by flow cytometry. (D) Confocal microscopy images of WT and RANKLΔCcl19 inguinal LNs, showing the subcapsular sinus area labelled for mCLCA1 (red) and RANKL (green). The RANKLΔCcl19 LN is devoid of RANKL expression. Representative of 4 mice. (E) Counterplot of LN LECs double stained for ITGA2b and RANK expression. Graph bar (n = 6) shows the percentage of LECs expressing both ITGA2b and RANK. (F) Histograms show RANK expression by LECs and BECs from skin and LNs. The percentage of ITGA2b+ cells is indicated. Graph shows their mean ± SD (n = 6) percentages. ns = not significant, *p<0.5, **p < 0.01, ***p < 0.001. (TIF) [file pone.0151848.s005.tif]
